# Auxin-Induced Plasma Membrane Depolarization Is Regulated by Auxin Transport and Not by AUXIN BINDING PROTEIN1

**DOI:** 10.3389/fpls.2018.01953

**Published:** 2019-01-17

**Authors:** Ivan A. Paponov, Julian Dindas, Elżbieta Król, Tatyana Friz, Vadym Budnyk, William Teale, Martina Paponov, Rainer Hedrich, Klaus Palme

**Affiliations:** ^1^ Faculty of Biology, Institute of Biology II/Molecular Plant Physiology, Albert-Ludwigs-University of Freiburg, Freiburg, Germany; ^2^ Norwegian Institute of Bioeconomy Research, Klepp, Norway; ^3^ Institute for Molecular Plant Physiology and Biophysics, University of Würzburg, Würzburg, Germany; ^4^ Renal Division, Department of Medicine, University Freiburg Medical Center, Freiburg, Germany; ^5^ Centre of Biological Systems Analysis and BIOSS Centre for Biological Signalling Studies, Albert-Ludwigs-University of Freiburg, Freiburg, Germany

**Keywords:** auxin, ABP1, plasma membrane depolarization, AUX1, endocytosis

## Abstract

Auxin is a molecule, which controls many aspects of plant development through both transcriptional and non-transcriptional signaling responses. AUXIN BINDING PROTEIN1 (ABP1) is a putative receptor for rapid non-transcriptional auxin-induced changes in plasma membrane depolarization and endocytosis rates. However, the mechanism of ABP1-mediated signaling is poorly understood. Here we show that membrane depolarization and endocytosis inhibition are ABP1-independent responses and that auxin-induced plasma membrane depolarization is instead dependent on the auxin influx carrier AUX1. AUX1 was itself not involved in the regulation of endocytosis. Auxin-dependent depolarization of the plasma membrane was also modulated by the auxin efflux carrier PIN2. These data establish a new connection between auxin transport and non-transcriptional auxin signaling.

## Introduction

In plants, indole-3-acetic acid (IAA), the most abundant naturally occurring auxin, plays a central role in orchestrating a wide range of context-dependent growth and developmental processes (Teale et al., 2006). Several auxin-binding proteins (ABPs) have been identified, some of which have been proposed to play a role in auxin perception (Jones, 1994). Of these, AUXIN BINDING PROTEIN1 (ABP1) has raised most interest because it binds several auxins, both natural and synthetic, in membrane fractions with affinities appropriate for a functional receptor (Dohrmann et al., 1978; Shimomura et al., 1986; Hesse et al., 1989). However, a conclusive demonstration of ABP1 as a genuine auxin receptor remains elusive. Early transgenic approaches, which increased or reduced expression of ABP1 using conditional ectopic expression or antisense approaches, did not reveal any *ABP1*-dependent phenotypes (Feckler, Palme, unpublished data). However, when it was reported in 2001 that a T-deoxyribonucleic acid insertion in *ABP1* caused embryo lethality (Chen et al., 2001), it seemed clear that ABP1 was the sole receptor for at least one crucial auxin-related process. This led to efforts in which weak alleles of *abp1* were isolated or transgenic lines were generated, in which ABP1 could be reversibly inactivated (David et al., 2007). These approaches generated data that uncovered a wide range of phenotypes, suggesting that the binding of auxin to ABP1 at the plasma membrane mediated changes in membrane polarization, the rate of cell expansion, the regulation of endocytosis, changes to microtubule organization, and activation of downstream signaling events (Braun et al., 2008; Robert et al., 2010). As evidence continued to accumulate, it became widely believed that highly localized, instantaneous ABP1-mediated auxin signaling events at the plasma membrane initiated non-transcriptional auxin-dependent signaling pathways. Although ABP1 contains a canonical KDEL motif at its C-terminus and is consequently retained in the ER (Campos et al., 1994), many authors have speculated on its role as a plasma membrane–localized auxin receptor (Sauer and Kleine-Vehn, 2011), but ABP1’s role in auxin signaling has remained controversial (Hertel, 1995; Habets and Offringa, 2015). Concerns were crystallized by recent findings in which *abp1* null alleles were indistinguishable from wild type plants, and the embryo lethality of Arabidopsis *abp1-1* was shown to be caused by the deletion of *BELAYAA SMERT (BSM)* and not by the disruption of *ABP1* (Dai et al., 2015). Most recently, a re-analysis of widely used ethanol-inducible *abp1* knock-down mutants showed that the phenotypes were caused by off-target effects (Michalko et al., 2016). To resolve the inconsistency between a lack of observable phenotype in qualified null *abp1* alleles (Gao et al., 2015) and strong rapid ABP1-dependent plasma membrane responses (Robert et al., 2010; Chen et al., 2014), we measured directly the role of ABP1 in the rapid auxin response.

In our previous work, we found that AUX1-mediated auxin transport is involved in auxin-induced plasma membrane depolarization (Dindas et al., 2018). However, we are yet to ascertain whether AUX1 is involved in the regulation of closely associated processes. Therefore, in this work, we investigated the effect of AUX1 in auxin-induced inhibition of endocytosis.

The involvement of AUX1-mediated auxin transport in the IAA-dependent regulation of plasma membrane potential raises the question of whether other auxin transport proteins also regulate auxin-dependent rapid plasma membrane responses. Among these proteins, PIN2 is an attractive candidate due to its epidermal localization and the agravitropic phenotype of *pin2* loss-of-function genotypes. Therefore, in this investigation, we tested whether auxin perception *via* PIN2 contributes to the plasma membrane depolarization response (Dindas et al., 2018).

This report re-evaluates the role of ABP1 at the plasma membrane and concludes that ABP1 makes no measurable contribution to the regulation of endocytosis or membrane depolarization. We also found that both AUX1 and PIN2 contributed to auxin-dependent depolarization of the plasma membrane.

## Materials and Methods

### Plant Material

Arabidopsis (*Arabidopsis thaliana*) seeds were surface sterilized for 10 min in 80% ethanol, 5% w/v calcium hypochlorite, and 0.1% Triton X-100. After three washes in 80% ethanol and one in 100% ethanol, seeds were left to dry under sterile conditions. Seeds were sown on plates containing 1% w/v sucrose, half-strength MS salts (Duchefa), and 15 g l^−1^ agar-agar (Merck) (pH 5.7). After stratification overnight at 4°C in darkness, plates were transferred to a growth chamber (16 h light/8 h darkness, 21°C, 100 μM m^−2^ s^−1^) for seed germination and maintained in a vertical position. In experiments with BFA treatments, 4-day old seedlings were incubated with 25 μM BFA (in the experiment with *abp1* mutants) for 90 min or with 50 μM BFA (in the experiments with *aux1* and *tir1*/*afb* mutants) dissolved in liquid 0.5 MS medium for 45 min or pre-treated with 10 μM 1-NAA (dissolved in liquid 0.5 MS medium) for 30 min followed by incubation with respective concentration of BFA and 10 μM 1-NAA. BFA stock solutions were made in DMSO up to a concentration of 50 mM. Control treatments contained an equal amount of DMSO. For electrophysiological experiments, Arabidopsis seedlings were grown sterile on 0.8–1% agarose supplemented with ¼-strength MS under controlled environmental conditions (12 h day vs. 12 h night; 21°C at day vs. 16°C at night; 120 μmol photons m^−2^ s^−1^) for 5 days. The following previously described lines of *Arabidopsis thaliana* Col-0, *aux1-7*, *aux1-22*, *eir1-1* (*pin2*), *tir1afb2afb3*, *abp1-c1*, and *abp1-TD1* lines have been used in this study.

### Experimental Setup for Intracellular Measurements

Sterile grown seedlings were exposed to standard bath solution (0.1 mM KCl, 1 mM CaCl_2_, 5 mM MES/BTP pH 5.5). Microelectrodes for impalement and preparation of application pipettes were pulled from borosilicate glass capillaries (Øout 1 mm, Øin 0.58 mm, w/filament, Hilgenberg, Germany) on a horizontal light amplification by stimulated emission of radiation puller (P2000, Sutter Instruments Co, USA). Microelectrodes were back-filled with 300 mM KCl and connected *via* an Ag/AgCl half-cell to a headstage (1 GΩ, HS-2A, Axon Inst., USA). The reference electrode was filled with 300 mM KCl as well. An IPA-2 amplifier (Applicable Electronics Inc., USA) and an NI USB-6259 interface (National Instruments, USA) were used for data collection. For application pipettes, the tips of microelectrodes were manually broken off to a 20–40 μm wide opening and back-filled with auxin-containing bath solution. Root hair cells of sterile grown *A. thaliana* seedlings were impaled under microscopic inspection (Axioskop, Zeiss, Germany) by using electronic micromanipulators (MM3A-LMP, Kleindiek Nanotechnik, Germany or Triple Axis Micromanipulator, Sensapex Oy, Finland). Application pipettes were also mounted on a micromanipulator (Triple Axis Micromanipulator, Sensapex Oy) and connected to a Picospritzer II microinjection dispense system (General Valve, USA) operating at 20 kPa to apply 1 s back pressure pulses. In cases where auxins were applied for longer periods of time, seedling roots were constantly perfused with bath solution (1 ml/min). In cases where application pipettes were applied, no perfusion was used.

### PIN Localization

For whole-mount immunolocalization of PIN1 and PIN2 in roots, 4-day-old Arabidopsis seedlings were fixed with 2% (w/v) paraformaldehyde (Fluka) and 0.4% Triton X-100 in microtubule-stabilizing buffer (MTSB) (pH 7.0) for 1 h and rinsed five times with MTSB/0.1% Triton X-100. Labeling was performed with rabbit anti-PIN1, guinea pig anti-PIN2 both at 1:500. Alexa Fluor 488-conjugated goat anti-rabbit and Alexa Fluor 555-anti-guinea pig secondary antibodies were used at 1:400 dilution. Solutions during the immunolocalization procedure were changed using a pipetting robot (Insitu Pro; Intavis).

### Microscopy and Image Analysis

Confocal images were taken using a Zeiss LSM 510 NLO confocal laser scanning microscope. Alexa Fluor 488 was excited by applying a 488 nm argon laser line in combination with a 500–550 band-pass filter. Alexa Fluor 555 was excited by applying a helium-neon 543 nm laser (HeNe laser) in conjunction with a 575-long pass filter. Quantitative analysis of confocal microscopic images was performed using Imaris 7.5.6 software (Bitplane AG). The fluorescence signal was detected using the “create surface” tool, and the fluorescence signal at the plasma membrane and in the BFA bodies was calculated. The level of signal internalization (the signal in the BFA bodies) was calculated as the ratio between an intensity of intracellular fluorescence signal and the intensity of total fluorescence signal expressed as a percentage. For every root, the estimation of the level of PIN internalization was based on 20–32 and 10–18 cells for PIN1 and PIN2, respectively. We used 5–10 roots for every treatment. Averages for every root were used for the calculation of standard deviation. Student’s *t* test was used for the evaluation of statistical significance between the experimental groups.

## Results

### ABP1 Is Not Involved in Auxin-Induced Plasma Membrane Depolarization

**Figure 1:**
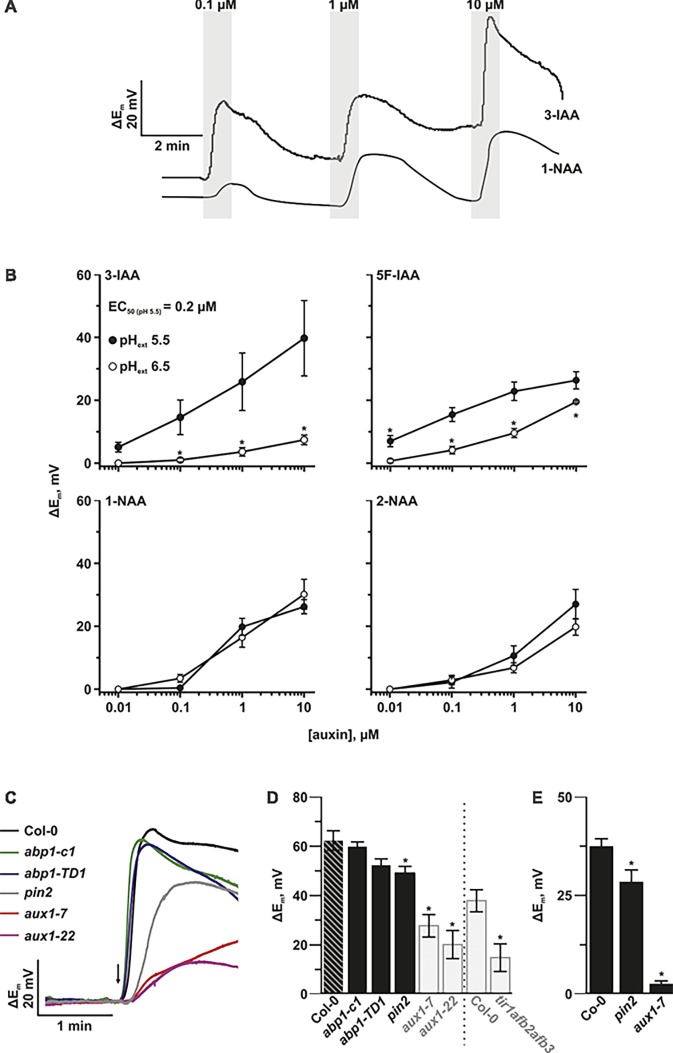
The auxin influx facilitator AUX1 is responsible for auxin-induced plasma membrane depolarization. **(A)** Representative voltage traces of the plasma membrane potential of *A. thaliana* root epidermal cells in response to the natural occurring auxin IAA and the synthetic analog 1-NAA. The gray bars show the periods of transient auxin treatment at the indicated concentrations. **(B)** pH-dependent amplitudes of plasma membrane depolarizations induced by IAA and the synthetic analogs 5F-IAA, 1-NAA, and 2-NAA. The response was tested at both pH values of 5.5 (closed circles) and 6.5 (open circles). Average values are shown. Error bars show standard error (*n* = 5–10). Asterisks mark significant differences to pH 5.5 (Student’s *t* test, *p* < 0.05). **(C)** Representative voltage traces of the plasma membrane potential of different *A. thaliana* mutant root hair cells in response to a 1 s pulse (arrow) of 10 μM IAA from a pressure operated application pipette. **(D)** Genotype-dependent amplitudes of root hair plasma membrane potential depolarization in response to a 1 s pulse of 10 μM IAA. *abp1-c1, abp1-TD1, aux1-7, aux1-22,* and *pin2* mutants were tested in the same experiment. The grey transparent bars highlight experiments for *aux1-7*, *aux1-22,* and *tir1afb2afb3* and their Col-0 controls that were already shown in our previous publication (Dindas et al., 2018) and are here included for comparison. The dashed line separates two independently performed experiments. Average values are shown. Error bars show SE (*n* = 10–11). Asterisks mark significant differences to Col-0 (Student’s *t* test, *p* < 0.05). **(E)** Genotype-dependent amplitudes of root hair plasma membrane potential depolarization. Bath solution was constantly exchanged. 10 μM IAA was applied *via* perfusion. *pin2* and *aux1-7* mutants were tested. Average values are shown. Error bars show SE (*n* = 7–15). Asterisks mark significant differences to Col-0 (Student’s *t* test, *p* < 0.05).

The lack of visible phenotypes in qualified null *abp1* alleles led us to revisit the question of whether depolarization of the plasma membrane and endocytosis are initiated by the binding of auxin to ABP1. Using voltage-recording single-barreled microelectrodes, we measured plasma membrane potential in *Arabidopsis thaliana* accession Col-0 root cells before and after the external application of indole-3-acetic acid (IAA) and selected synthetic auxins (Figures 1A,B). In line with Dindas et al. (2018), resting membrane potentials were in the range of −150 mV. Plasma membrane potential depolarization was pH dependent after application of the active auxins IAA and 5F-IAA with depolarization being observed at acidic external pH (Figure 1B). In the case of IAA, strong depolarization of epidermal cell membranes was seen from 100 nM with an EC_50_ of 200 nM (Figure 1B). In contrast, the synthetic auxin analog 1-NAA and its non-auxinic isomer 2-NAA did not induce comparable depolarizations at concentrations <1 μM (Dindas et al., 2018). In order to test whether ABP1-mediated auxin-induced depolarization, we compared the plasma membrane potential of wild type and recently generated *abp1* knockout mutants (Gao et al., 2015). Impalement of root hair cells with voltage-recording microelectrodes revealed that locally applied short pulses of IAA caused an instant membrane depolarization of up to 76 mV in wild-type root hairs (Figure 1C). Membrane depolarization induced by pulses of IAA in plants homozygous for the *abp1-c1* mutant allele (a full knock-out of the *ABP1* gene) was indistinguishable from wild type. This result was corroborated with an independent null mutant allele *abp1-TD* (Gao et al., 2015). Figure 1D shows no significant changes in the absolute values of auxin-induced plasma membrane depolarization in all *ABP1* knockout mutants tested. We therefore conclude that ABP1 does not mediate auxin-induced membrane potential depolarization.

### Auxin Transport Is Essential for Auxin-Induced Plasma Membrane Depolarization

Several families of plasma membrane localized proteins also specifically bind and transport auxin, with at least one, AUX1, mediating auxin-induced depolarization. In the current study, we investigated the additional auxin transport mutant, *pin2* to understand whether members of the PIN family of auxin transporters are also able to affect auxin-induced plasma membrane depolarization. As we reported previously (Dindas et al., 2018), auxin-dependent depolarization was strongly altered in the two tested *aux1* mutants, as well as in the *tir1afb2afb3* auxin-receptor mutant (Figures 1D,E). We therefore conclude that AUX1, and not ABP1, acts as an auxin-dependent signaling platform for plasma membrane depolarization. Auxin-induced plasma membrane depolarization was also decreased in *pin2* seedlings, which indicates that this response might be related to the regulation of auxin concentration in the cell (Figure 1E).

### Inhibition of Endocytosis Does Not Depend on AUX1 or ABP1

**Figure 2:**
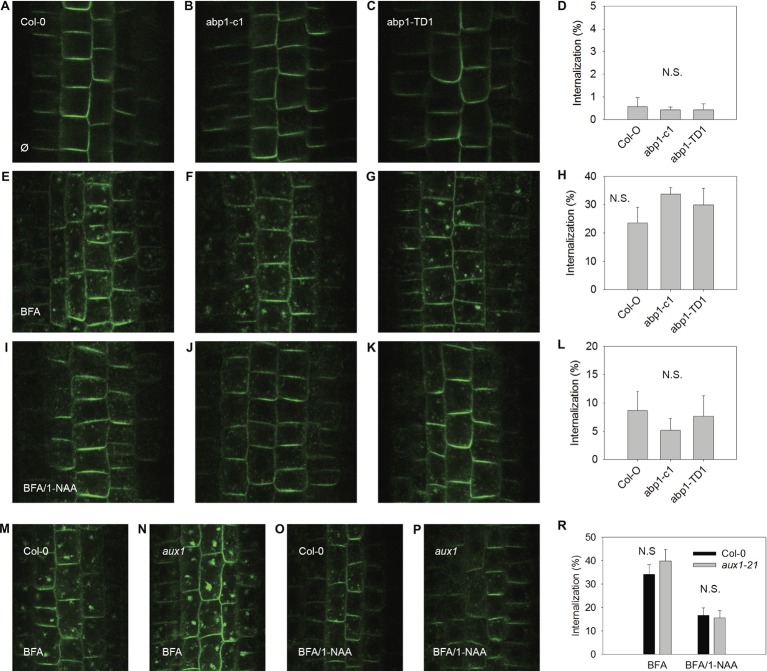
BFA-induced PIN1 internalization and its regulation by NAA are independent of ABP1 and AUX1. PIN1 localization under control conditions in *A. thaliana* wild type (accession Col-0) **(A)**, and mutant lines *abp1-c1* **(B)**, *abp1-TD1* **(C)**; percentage of PIN1 internalization under control conditions **(D)**; BFA-induced PIN1 internalization (25 μM BFA) in Col-0 **(E)**, *abp1-c1* **(F)**, *abp1-TD1* **(G)**; percentage of PIN1 internalization after BFA treatment **(H)**; 1-NAA (10 μM) inhibits BFA-induced PIN1 internalization in Col-0 **(I)**, *abp1-c1* **(J)**, *abp1-TD1* **(K)**; percentage of BFA-induced PIN1 internalization inhibited by 1-NAA **(L)**; PIN1 localization after BFA treatment in Col-0 **(M)** and *aux1* **(N)**. PIN1 localization after pretreatment with 1-NAA and consequent treatment with 50 μM BFA supplemented with 1-NAA (BFA/1-NAA) in Col-0 **(O)** and *aux1* **(P)**. Percentage of BFA-induced internalization in Col-0 and *aux1* after BFA and BFA/1-NAA treatments **(R)**. Bars show the mean and ±SD of a parameter reflecting PIN internalization, which was evaluated as a percentage of an intensity of internal fluorescence to the whole fluorescence intensity of a cell. Statistical significance was done using Student’s test. Letters indicate significant differences *p* < 0.05. N.S. indicates insignificant differences *p* < 0.05.

**Figure 3:**
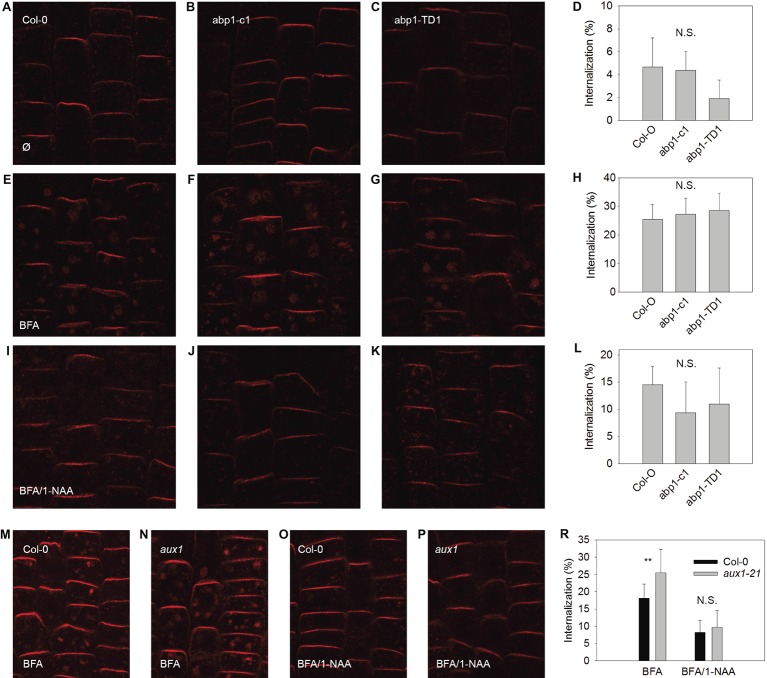
BFA-induced PIN2 internalization and its regulation by NAA are independent of ABP1 and AUX1. PIN2 localization under control conditions for Col-0 **(A)**, abp1-c1 **(B)**, abp1-TD1 **(C)**; percentage of PIN2 internalization under control conditions **(D)**; BFA-induced PIN2 internalization (25 μM BFA) in Col-0 **(E)**, abp1-c1 **(F)**, abp1-TD1 **(G)**; percentage of PIN2 internalization after BFA treatment **(H)**; 1-NAA (10 μM) inhibits BFA-induced PIN2 internalization in Col-0 **(I)**, abp1-c1 **(J)**, abp1-TD1 **(K)**; percentage of BFA-induced PIN2 internalization inhibited by 1-NAA **(L)**. PIN2 localization after BFA treatment in Col-0 **(M)** and aux1 **(N)**. PIN2 localization after pretreatment with 1-NAA and consequent treatment with 50 μM BFA supplemented with 1-NAA (BFA/1-NAA) in Col-0 **(O)** and *aux1* **(P)**. Percentage of BFA-induced PIN2 internalization in Col-0 and *aux1* after BFA and BFA/1-NAA treatments **(R)**. Bars show the mean and ±SD of a parameter reflecting PIN2 internalization, which was evaluated as a percentage of an intensity of internal fluorescence to the whole fluorescence intensity of a cell. Statistical significance was done using Student’s test. ** indicate significant differences at *p* < 0.01. N.S. indicates non-statistically significant differences at *p* < 0.05.

In order to test the scope of AUX1 and ABP1-related influences over auxin-dependent physiological processes at the plasma membrane, we applied an established assay for testing the effects of synthetic auxin analogs 1-NAA on endocytosis (Figures 2, 3). After exposure to BFA (a reversible inhibitor of exocytosis) treatments, PIN1 and PIN2 (markers of plasma membrane protein internalization) accumulated in large intracellular compartments in wild-type seedlings (Figures 2E,M, 3E,M), indicating endocytic internalization of PIN1 and PIN2. Pretreatment with 10 μM 1-NAA significantly inhibited BFA-induced PIN1 and PIN2 internalization (Figures 2H,I,L,O,R, 3H,I,L,O,R). As AUX1 was involved in rapid membrane depolarization, we next tested the effects of 1-NAA in *aux1* and found that 1-NAA decreased BFA-induced PIN internalization to a similar extent as was observed in wild-type roots (Figures 2O,P,R, 3O,P,R). We therefore conclude that the inhibitory effect of 1-NAA on PIN internalization is not mediated by AUX1. After analysis of the 1-NAA-induced PIN internalization response in *abp1-c1* and *abp1-TD* null mutant alleles, we observed similar degrees of PIN internalization in roots of both genotypes and wild type (Figures 2J–L, 3J–L), showing that BFA-induced PIN internalization is also independent of ABP1.

## Discussion

### Plasma Membrane Depolarization Does Not Depend on ABP1

It has long been hypothesized that ABP1 is an extracellular auxin receptor, which mediates electric responses at the plasma membrane (Barbier-Brygoo et al., 1989; Leblanc et al., 1999). However, this function has never been directly shown. Characterization of two new lines of *abp1* mutants has recently downgraded the significance of ABP1 for plant growth and development. Despite this, the possibility that ABP1 is involved in electric responses at the plasma membrane has never been ruled out. Using *abp1* loss-of function lines, we can now do just this, by showing that ABP1 is not involved in auxin-dependent plasma membrane depolarization (Figures 1C,D).

### Dependence of Auxin-Induced Depolarization on Auxin Transport and Growth

Auxin-induced depolarization is dependent on auxin transport as auxin-dependent membrane depolarization is reduced in both influx and efflux carrier mutants, *aux1* and *pin2* (Figures 1C–E). The impact of the influx carrier was much higher, as depolarization was almost completely absent in *aux1* mutants compared to a reduction of 20% in the *pin2* mutant when compared to wild type. Both auxin transporters contribute to auxin accumulation in epidermis cells; the mechanism of action is through auxin-induced cytoplasmic Ca^2+^ transient concentration maxima, which require SCF^TIR1/AFB^-based auxin signaling (Dindas et al., 2018).

The depolarization induced by IAA is likely to be auxin specific and physiologically relevant. This conclusion is supported by the correlation between the activity of auxins on membrane depolarization and their activity on inhibition of root growth: the most potent auxin IAA with respect to membrane depolarization caused the strongest inhibition of root growth (Rahman et al., 2007). Second, the dose responses with respect to membrane depolarization and root growth are similar: the minimal concentration sufficient to affect both processes is about 0.1 μM with saturation of response for both processes at 10 μM.

### Both AUX1 and ABP1 Are Not Involved in Mediating Endocytosis

Endocytosis was not affected in *aux1* (Figures 2, 3), indicating that its regulation is independent of the auxin-related regulation of plasma membrane potential. The hypothesis that an auxin-ABP1 interaction initiates a signal, which regulates endocytosis, is based on an analysis of conditional immune-modulation and antisense *abp1* knockdown lines (Robert et al., 2010). However, the phenotypes of these lines have been attributed to off-target effects (Dai et al., 2015). The re-evaluation of this effect on qualified null *abp1* allele lines was therefore necessary. Our measurements show that ABP1 does not regulate endocytosis (Figures 2, 3).

### Two Rapid Auxin Responses: Plasma Membrane Depolarization and Endocytosis Are Independent

Both membrane depolarization and the inhibition of endocytosis are rapid auxin-induced responses. However, pharmacological and genetic experiments have shown that they are independent. The conclusion can be made as 1) auxin-induced membrane depolarization has a higher sensitivity to IAA than 1-NAA, whereas endocytosis is more sensitive to 1-NAA than to IAA (Paciorek et al., 2005); 2) membrane depolarization and the inhibition of endocytosis are also different in their speed of response: after the application of auxin, the plasma membrane depolarizes instantaneously, but endocytosis is only halted after 5 min (Dindas et al., 2018; Robert et al., 2010); and 3) auxin-induced depolarization was strongly reduced in the *aux1* mutant, whereas the inhibition of endocytosis was unaffected. It is important to mention that to test the dependence of endocytosis inhibition by auxin from AUX1, we used 1-NAA because this is one of the most active auxins at inhibiting endocytosis (Paciorek et al., 2005; Robert et al., 2010). However, 1-NAA is able to diffuse through membranes in the absence of the AUX1 transporter (Yamamoto and Yamamoto, 1998); thus, we expected that 1-NAA-induced inhibition of endocytosis would be independent of AUX1 function. In contrast to 1-NAA, the natural auxin IAA without the addition of antioxidants has very low activity in terms of inhibition of endocytosis in the wild type, as the minimal concentration sufficient to inhibit endocytosis is about 1,000 times higher than the IAA concentration sufficient to modulate membrane depolarization (100 and 0.1 μM for inhibition of endocytosis and membrane depolarization, respectively) (Paciorek et al., 2005 and our data). This, in itself, indicates different mechanisms of regulation of endocytosis and membrane depolarization by IAA.

The data reported here clarify an important and intensively discussed point regarding the mechanisms of a plant’s non-transcriptional responses to auxin. We show that the long observed but poorly understood IAA-induced plasma membrane depolarization does not depend on ABP1 but instead is due to the co-transport of H^+^ and IAA into the cell by plasma membrane localized AUX1 rather than ABP1. Furthermore, our results show that neither AUX1 nor ABP1 plays roles in mediating endocytosis, another non-transcriptional auxin response. Given that ABP1 seems not to fulfill the functions, it was assigned in auxin action initially, and future studies need to find the real function of ER auxin-binding protein in plant growth and development.

## Author Contributions

IP and KP conceived the project. IP, KP, JD, EK, WT, and RH wrote the manuscript. JD, EK, RH, IP, TF, VB, and MP performed the experiments and analyzed the data.

### Conflict of Interest Statement

The authors declare that the research was conducted in the absence of any commercial or financial relationships that could be construed as a potential conflict of interest.
